# Effectiveness of behaviour change techniques in physiotherapy interventions to promote physical activity adherence in lower limb osteoarthritis patients: A systematic review

**DOI:** 10.1371/journal.pone.0219482

**Published:** 2019-07-10

**Authors:** Matthew Willett, Joan Duda, Sally Fenton, Charlotte Gautrey, Carolyn Greig, Alison Rushton

**Affiliations:** 1 Centre of Precision Rehabilitation for Spinal Pain (CPR Spine), School of Sport, Exercise and Rehabilitation Sciences, University of Birmingham, Birmingham, United Kingdom; 2 School of Sport, Exercise and Rehabilitation Sciences, University of Birmingham, Birmingham, United Kingdom; 3 MRC-Arthritis Research UK Centre for Musculoskeletal Ageing Research, University of Birmingham, Birmingham, United Kingdom; 4 School of Life and Medical Sciences, University of Hertfordshire, Hatfield, United Kingdom; EHESP Paris, FRANCE

## Abstract

**Background:**

Lower limb osteoarthritis (OA) causes high levels of individual pain and disability and is an increasing socio-economic burden to global healthcare systems. Physical Activity interventions are commonly provided by physiotherapists to help patients with lower limb OA manage their clinical symptoms.

**Objective:**

To identify and evaluate the effectiveness of behavioural change techniques (BCTs) within physiotherapy interventions to increase physical activity (PA) adherence in patients with lower limb OA.

**Design:**

A systematic review was conducted, following Cochrane guidelines according to a published and registered protocol (CRD42016039932). Two independent researchers conducted searches, determined eligibility, assessed risk of bias (Cochrane tool), intervention fidelity (NIHBCC checklist), and coded randomised controlled trials (RCTs) for BCTs (V1 taxonomy). BCT effectiveness ratios were calculated and RCT risk of bias and intervention fidelity were summarised narratively.

**Data sources:**

A highly sensitive search strategy was conducted on Medline, Embase, PsycINFO, CENTRAL, CINAHL and PEDro and grey literature databases from inception to January 2^nd^, 2018. Reference lists of included RCTs and relevant articles were reviewed, and a citation search was conducted using Web of Science.

**Eligibility criteria for selecting studies:**

RCTs that evaluated the effectiveness of a physiotherapy intervention that incorporated ≥1 BCT that promoted home or community-based PA adherence in patients with lower limb osteoarthritis.

**Results:**

Twenty-four RCTs (n = 2366 participants) of variable risk of bias (RoB) (5 low; 7 moderate; 12 high) and poor intervention reporting from 10 countries were included. Heterogeneity of intervention BCTs and PA adherence outcome measures precluded meta-analysis. Thirty-one distinct BCTs were identified in 31 interventions across RCTs. In general, BCTs demonstrated higher effectiveness ratios for short-term and long-term PA adherence compared with medium-term outcomes. The BCTs ‘behavioural contract’, ‘non-specific reward’, ‘patient-led goal setting’ (behaviour), ‘self-monitoring of behaviour’, and ‘social support (unspecified) demonstrated the highest effectiveness ratios across time points to promote PA adherence.

**Conclusions:**

BCTs demonstrate higher short and long-term than medium-term effectiveness ratios. Further research involving low RoB RCTs incorporating transparently reported interventions with pre-specified BCTs aimed at optimising lower limb OA patient PA adherence is required.

## Introduction

Osteoarthritis (OA) is a progressive degenerative disease associated with a loss of local articular cartilage, a local inflammatory response, and contiguous bone remodelling [1]. In the United Kingdom, OA is the primary cause of individual level disability and it contributes substantially to global health care costs [1]. The hip and knee are the primary synovial joints effected by OA, with a point prevalence of 11% and 24% respectively [2]. Patients with lower limb (hip and knee) OA may experience severe pain and disability, and a loss of function and psychological well-being [3,4]. As no known cure for OA exists [1], and the population ages, the global point prevalence of lower limb OA is likely to increase further [2].

Interventions that incorporate exercise are recommended first forms of non-pharmacological treatment to aid patients with lower limb OA to manage their symptoms [1]. Physiotherapists are the primary health professionals responsible for providing exercise interventions to patients with lower limb OA in several countries [5]. Although exercise interventions improve short-term clinical outcomes, such as reduced pain and increased function, in patients with lower limb OA, these positive effects are generally not maintained at medium (6 months) to long-term (≥12 months) follow up assessments [3,4]. This could be due to several factors including limited long-term effectiveness of the prescribed intervention, progression of OA disease severity, or a gradual reduction in patient adherence to health professional recommendations and an inability to maintain changes in their exercise behaviours [6].

To experience exercise related health benefits, patients need to maintain their activity level for a sustained period of time [7]. However, patients with OA are less active than non-symptomatic populations [8], and perceive that exercise is unsafe and that they require greater support to maintain their physical health, which likely impacts on their exercise adherence [9].

Prolonged physical inactivity has been associated with several reductions in the general health in patients with lower limb OA including the development of comorbidities such as obesity, diabetes, and heart disease [10], declining function and elevated pain levels, and an acceleration of the underlying OA disease process [11]. Exercise is a planned and structured physical activity (PA) aimed at improving or maintaining physical fitness [12]. PA encompasses structured exercise as well as broader concepts such as leisure activity which could be important at improving lifestyle factors beyond clinical outcomes, such as patient well-being [12]. Furthermore, maintaining prescribed exercise levels requires greater patient effort and behaviour change than optimising more general PA behaviours [13]. Therefore, it is important for physiotherapist interventions to incorporate behavioural techniques which target adherence to PA broadly, in addition to specific exercises. However, knowledge of the most effective techniques to optimise patient adherence to PA behaviours is currently lacking [14].

Behaviour change techniques (BCTs) are the smallest active components of interventions which are designed to optimise patient health behaviours [15]. However, identifying BCTs within research has proved problematic due to inconsistent reporting of behaviour change interventions [15]. The Behaviour Change Taxonomy V1 [15] was developed by researchers with expertise in behaviour change to enable for consistent reporting and increased clarity when identifying BCTs within RCT interventions. The taxonomy contains 93 distinct BCTs grouped into 16 hierarchies [15] and has been used extensively in the behaviour change literature.

Systematic reviews have examined the effectiveness of BCTs on PA in people with diabetes [16], cardiac conditions [17], older adults [18,19], rheumatoid arthritis [20], obesity [21,22], and broader patient populations [23–26]. In these reviews goal setting, problem solving, action planning, feedback on behaviour, self-monitoring of behaviour, instruction in how to perform the behaviour, demonstration of the behaviour, and behavioural practice/rehearsal have been commonly identified BCTs that increase patient PA.

To date, two systematic reviews [27,28] have reported on BCT use in physiotherapy interventions on musculoskeletal pain populations. However, these reviews did not measure individual BCT effectiveness and therefore could not make evidence-based recommendations of their use in clinical practice. Furthermore, these reviews focused on broader patient groups and their results may not be specific to lower limb OA patients, and they did not search the grey literature, meaning that up to 10% of relevant literature could have been missed [29]. Keogh et al., (2015) [27] identified 33 BCTs within physiotherapy led group-classes. Patients with lower limb OA are most commonly treated individually (1 to 1) in clinical practice [30] as tailoring the intervention to a patients specific presentation is fundamental to optimising PA adherence [31] and attendance levels are generally higher at individual consultations than group classes [32]. Kunstler et al., (2017) identified 30 BCTs across physiotherapy RCT interventions that aimed to optimise PA. However only one researcher was involved in BCT coding and the authors did not outline any training that was undertaken to enable accurate identification of intervention BCTs. Training is recommended prior to using the V1 taxonomy [15], and reduced inter-rater reliability has been measured in systematic reviews where coders have not undergone the prescribed training (Kappa = 0.55) [33], compared with those that have (Kappa range 0.68–0.92) [18,20,22–24,34].Therefore, the accuracy of BCT identification within this review cannot be verified.

Therefore, the primary objective of this systematic review was to identify the BCTs used in individual (1 to 1) physiotherapy interventions to promote PA adherence beyond the clinic (home and community) in lower limb OA patients. The secondary objective was to evaluate the effectiveness of individual BCTs at optimising PA adherence to make evidence-based recommendations to aid effective management of patients with lower limb OA.

## Methods

A systematic review was conducted according to a registered (CRD42016039932) and published protocol [35] using methodological guidelines from the Cochrane Collaboration’s Musculoskeletal group [36] and Centre of Reviews and Dissemination (CRD) [37]; and is reported in line with the Preferred Reporting Items for Systematic Reviews and Meta-Analyses statement (PRISMA) [38].

### Eligibility criteria

The eligibility criteria are detailed in Table 1.

**Table 1:** Eligibility criteria.

Population	Adult participants (≥18 years) with lower limb OA (hip and/or knee), diagnosis through self-report of symptoms or imaging [2]. RCTs including participants with other joint related pathologies (e.g. rheumatoid arthritis, hemochromatosis) that contributed to ≥25% of the population, or participants who were awaiting, or had undergone joint surgery for treatment of OA symptoms were excluded.
**Interventions**	Land based outpatient intervention where a physiotherapist delivered the intervention individually (1 to 1) to the patient. Although others (e.g. healthcare professionals/ spouses) could be involved in the intervention’s delivery, the physiotherapist had to be the primary healthcare provider and their role needed to be clearly established. The intervention had to include at least one BCT specifically enhancing PA adherence away from the clinic e.g. activity diary/pedometer or follow up care (phone calls) that made it unique from its comparator group (as defined by the V1 taxonomy) to enable measurement of its effectiveness.
**Comparisons**	Other ‘active’ or ‘placebo’ interventions, ‘no treatment’ or ‘usual care’
**Outcomes**	Primary Outcomes: Physical Activity or Adherence measures Secondary Outcomes: Pain, Function, Quality of Life, Self-Efficacy, Any adverse events
**Trial design**	Randomised controlled trials (RCTs)
**Language**	Written in, or translated into English

### Information sources and searches

Two independent researchers (MW, ChG) conducted a highly sensitive search strategy, developed in collaboration with a subject specific librarian, on the following sources from inception up to January 2^nd^ 2018:

- Databases: Medline (OVID), EMBASE, Cumulative Index to Nursing and Allied Health Literature (CINAHL), the Cochrane Register of Controlled Trials (CENTRAL), PsycInfo (OVID) and the Physiotherapy Evidence Data base (PEDro).
- Grey Literature: ‘ZETOC’ and ‘Conference Proceedings Citation Index’.
- Trial registries: The ‘clinicaltrial.gov’ and World Health Organisation’s (WHO) trial portal

The search strategy was developed by incorporating terms from previous systematic reviews on participants with lower limb OA, who were treated with behavioural, and physiotherapy interventions. Additional keywords were added from RCTs identified in the scoping search undertaken prior to the review [39,40], and filters recommended by the Cochrane Collaboration. The strategy was adapted for each database, and the Medline (OVID) search strategy is included below.

#### Example search strategy: Medline (Ovid) Search Strategy: 2^nd^ January 2018

1. exp osteoarthritis/
2. osteoarthr$.tw.
3. (degenerative adj2 arthritis).tw.
4. arthrosis.tw.
5. Or/ 1–4
6. knee/
7. exp knee Joint/
8. knee$.tw.
9. hip/
10. exp hip joint/
11. hip$.tw.
12. Or/ 6–11
13. exp Self Care/
14. ((self or symptom$) adj (care or help or manag$ or directed or monitor$ or efficacy or admin$)).tw.
15. Patient Education as Topic/
16. ((health or patient$) adj2 (educat$ or information)).tw.
17. exp Consumer Participation/
18. ((patient$ or consumer$) adj part$).tw.
19. "Power (Psychology)"/
20. empower$.tw.
21. Holistic Health/
22. (holistic or wholistic).tw.
23. "activities of daily living"/
24. (activit$ adj2 daily adj living).tw
25. social support/
26. (social adj (support or network$)).tw.
27. (support adj system$).tw.
28. exp Adaptation, Psychological/
29. (psychologic$ adj (adjust$ or adapt$)).tw.
30. (cope or copes or coping).tw.
31. exp Behavior Therapy/ or exp cognitive therapy/ or self manage$.ti.
32. (adapt$ adj behav$).tw.
33. (behav$ adj (therap$ or intervention$)).tw.
34. health education/ or self efficacy/ or Exercise/ or health behavior/
35. compliance/ or patient compliance/
36. conditioning, operant/
37. exp "Reinforcement (Psychology)"/
38. operant conditoning.mp.
39. respondent treatment.mp.
40. relaxation.mp. or exp Relaxation/
41. graded activity.mp.
42. health promotion/
43. (psycholog* technique or behavio?r technique).mp.
44. behavio?r Change.mp.
45. self efficacy.mp.
46. Motivation/ or motivation*.mp.
47. primary prevention/
48. Psychology.mp. or Psychology/
49. Adherence.mp.
50. Or/ 13–49
51. exp Physical Therapy Modalities/
52. physiotherap$.tw.
53. (physiotherap$ or physical therap$ or pt).mp.
54. physiotherap$.mp.
55. kinesiotherap$.tw.
56. exp Rehabilitation/
57. rehab$.tw
58. Physical Activity.mp.
59. Or/ 51–58
60. randomi?ed controlled trial.pt.
61. controlled clinical trial.pt.
62. randomi?ed.ab.
63. placebo.ab.
64. drug therapy.fs.
65. randomly.ab.
66. trial.ab.
67. groups.ab.
68. Or/ 60–67
69. exp animals/ not humans.sh.
70. 68 not 69
71. 5 and 12 and 50 and 59 and 70

### Study selection

Two independent researchers (MW, ChG) reviewed the titles and abstracts generated by the initial searches and then obtained full text copies of those RCTs which appeared appropriate for inclusion, or where doubt remained. The same two researchers then conducted a citation search using the ‘Web of Science’ database (included RCTs) and reviewed the reference lists (included RCTs and relevant reviews) to identify further RCTs. At each stage, if the two researchers could not agree on the eligibility of an RCT, a third researcher was asked to mediate the final decision (AR).

### Data collection process and items

A data extraction sheet was piloted, adjusted, and subsequently used by two independent researchers (MW, SF) to record information on RCT participants, interventions, and characteristics of those delivering the BCTs including any training they undertook. Data obtained for PA, adherence, pain, function, quality of life, self-efficacy, and adverse effects outcome measures were also extracted. An analysis of contemporary related systematic reviews showed large variation in definitions of short, medium, and long-term follow up periods. Therefore, outcome timings of included RCTs were reviewed by two independent researchers (MW, SF) and short, medium, and long-term time points were determined after data extraction, to more accurately reflect the body of literature. Any disagreement in reporting was handled through researcher discussion. If agreement could not be reached, a third researcher with expertise in systematic review methodology (AR) or behaviour change (JD) was asked to mediate. Authors were emailed if data were missing or unclear, and intervention manuals were sought if they were not included in either RCT publications or as supplementary materials to aid further coding of BCTs.

### Risk of bias (RoB) and fidelity assessment in individual RCTs

Two independent researchers (MW, SF) piloted RCT RoB and intervention fidelity assessments to clarify understanding and increase consistency. They subsequently assessed RoB and intervention fidelity of all included RCTs. RCT RoB was assessed using the Cochrane risk of bias tool across six domains, with each domain judged as either ‘Low’, ‘High’, or ‘Unclear’ [41]. The tool was developed from evidence from empirical studies [41] and has been used extensively in contemporary systematic reviews. RCT intervention fidelity was assessed using the National Institutes of Health Behavioural Change Consortium’s (NIHBCC) checklist which has demonstrated validity and reliability and was developed specifically for assessing behavioural change interventions [42]. The checklist contains 40 components across 5 domains [43]. Each domain was graded as either ‘present’ or ‘absent’ depending on the number of individual components which were clearly reported, which were in turn judged as ‘present’, ‘absent’ or ‘unclear’. Any disagreements over RCT RoB or fidelity were resolved by researcher consensus. If consensus could not be reached, a third researcher with expertise in internal validity (AR) or fidelity (JD) respectively, was asked to mediate the decision. Full justification of RoB and fidelity assessment tools and their use can be found in the protocol for this systematic review [35].

### BCT coding

RCT interventions were coded for BCTs using Michie and colleagues' V1 behaviour change taxonomy [15] by two independent researchers (MW, SF) following training in the taxonomy’s use [44]. Both the behavioural physiotherapy (i.e. the physiotherapy intervention that contained a BCT specifically targeting PA adherence) and any active comparator group, including other forms of physiotherapy, were coded for BCTs. The taxonomy was piloted on several RCTs a priori and the researchers met regularly to maintain understanding and minimise any post-learning effect during coding [45]. Any disagreement between reviewers was resolved through discussion. If agreement could not be reached, a third reviewer (JD) was asked to mediate. BCT coding agreement between researchers was calculated using Cohen’s Kappa Statistic [46].

Pilot BCT coding highlighted several problems with the V1 taxonomy’s use which led to the following alterations.

1. The BCTs 1.1 goal setting (behaviour) and 1.3 goal setting (outcome) were subdivided into 1.1a (patient-led) and 1.1b (therapist-led). Goal setting has a very broad definition in the taxonomy (‘set or agree on a goal defined in terms of the behaviour/outcome to be achieved’) [15]. Evidence suggests that goals that are decided by the patient (patient-led) lead to greater engagement and achievement than goals which are set by the therapist (therapist-led) [47].
2. The BCTs 2.1 monitoring of others without feedback, 2.3 self-monitoring of behaviour, 2.5 monitoring of outcomes of behaviour without feedback, and 3.1 social support (unspecified) were subdivided into ‘a’ (reported as a technique within the intervention), and ‘b’ (reported as an outcome measure). The taxonomy does not code BCTs which are data collection only processes [48]. However, due to the behaviour of interest, several RCTs included a monitoring device (e.g. exercise diary/follow up telephone call), which were not necessarily an explicit intervention component but would likely increase PA adherence.

### Measurement of analysis and summary measures

BCT use within and across RCTs and their associated taxonomy hierarchies were tabulated (Objective 1). The initial scoping search, which was conducted prior to the systematic review, suggested there would be considerable heterogeneity of intervention BCTs and PA adherence measures and therefore, meta-analysis and meta-bias assessment would not be appropriate. Therefore, effect sizes were used to determine intervention effectiveness and enable comparison between RCTs to inform the narrative synthesis. Effect sizes of PA and/or adherence outcomes were calculated using the standardised mean difference (SMD) and probability ratio (PR) for continuous and dichotomous data respectively [49]. The term ‘probability’ was used in favour of ‘risk’ as in this review optimisation of PA adherence was desirable [49]. For dichotomous data, an event was considered to have occurred when a participant achieved their PA or adherence goals to the levels stated in the associated RCT. It was calculated by dividing the number of times the PA or adherence level was achieved by the number of participants in a group [49]. Effect sizes where confidence intervals did not pass 0 for continuous outcomes (negative values favouring behavioural physiotherapy intervention) or 1 for dichotomous (> 1 favouring the behavioural physiotherapy intervention) were considered statistically significant. To enable inclusion of data from RCTs that contained more than one intervention or comparator group, data were combined into a single pairwise comparison using methods suggested by the Cochrane collaboration when groups were considered sufficiently homogenous [50]. 95% confidence intervals were calculated, and significance was set at *p* <0.05. Due to outcome heterogeneity, RCT outcomes were grouped into self-report measures of adherence and PA and direct measures of PA domains respectively. Although all PA and adherence data were extracted where available, if more than one outcome was reported within a domain, the effect size was calculated using summary measures of PA [26]. Due to the focus of the systematic review, PA or adherence to physiotherapist recommendations was used to calculate effect sizes if total PA or adherence data were not available.

### BCT effectiveness

As meta-analysis was not anticipated, meta-regression could not be incorporated into the analysis to determine the effectiveness of BCTs. Therefore, the ‘effectiveness ratio’ was used to determine the effect of each BCT on optimising PA adherence [51]. The effectiveness ratio offers a simple, user friendly technique for interpreting the effectiveness of individual BCTs by comparing effective interventions with ineffective interventions [51], and has been used in several contemporary systematic reviews published in peer review journals. It was calculated by dividing the number of times the BCT was identified in behavioural physiotherapy interventions that demonstrated a favourable significant effect (as measured by SMD and PR effect sizes) by the number of times it was coded across all interventions [51] (Objective 2). BCT effectiveness ratios were calculated at short, medium, and long-term time points to evaluate whether they changed over time. To ensure that BCT effectiveness ratios reflected the active ingredients within interventions that optimized PA adherence, only those techniques unique to the behavioural physiotherapy intervention (when compared to active comparator and control interventions) were used when calculating BCT effectiveness ratios [52].

## Results

### RCT Selection

**Fig 1:**
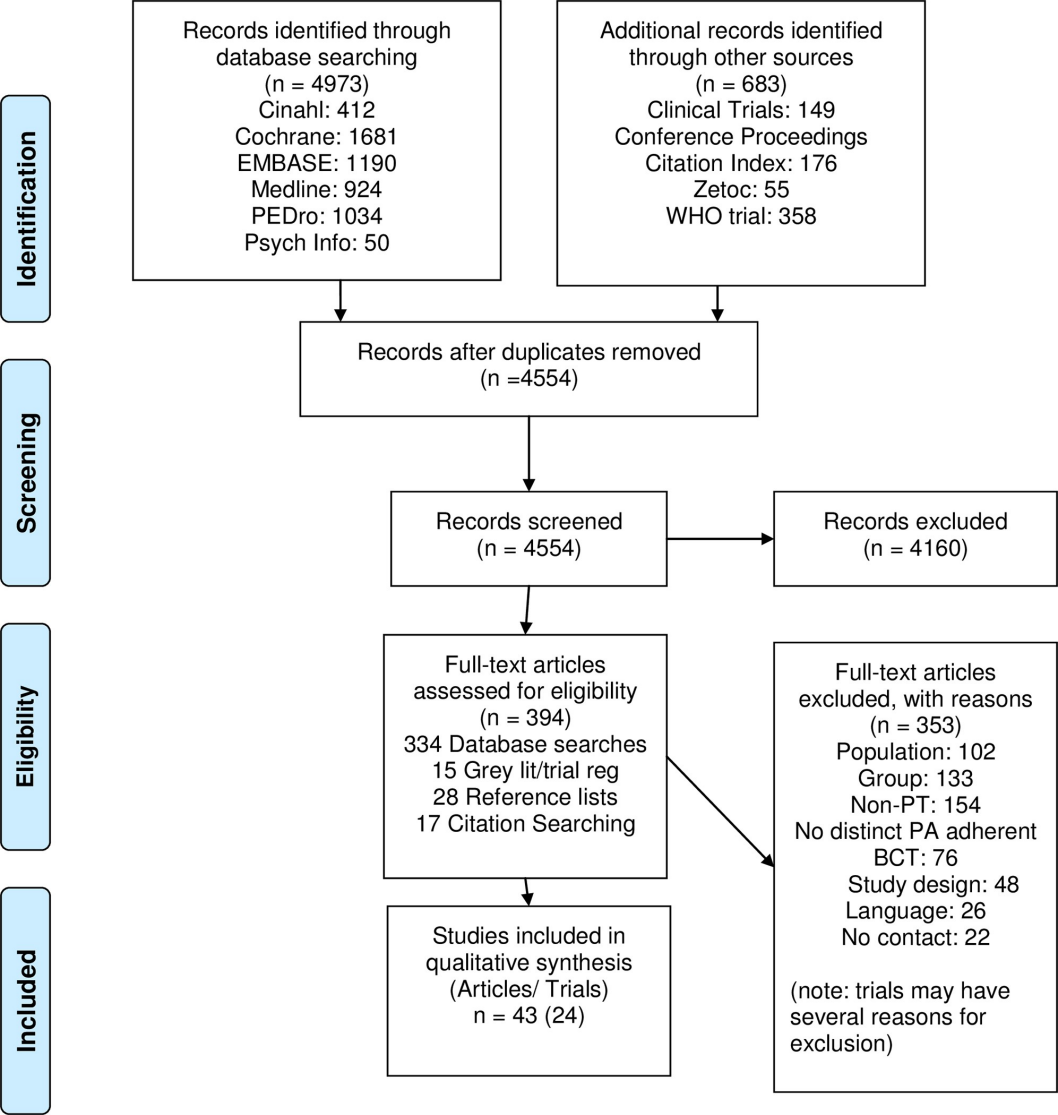
RCT selection flow diagram.

Initial searches retrieved 5656 references with 4554 titles and abstracts once duplicates were removed. 394 full text articles were reviewed and 24 RCTs from 43 articles were included. At the full text stage, articles were excluded because RCT interventions were group based (n = 133), not conducted by a physiotherapist (n = 154) or did not contain an appropriate or unique BCT (n = 76). Additional reasons for RCT exclusion were; alternative populations (n = 102), non-RCT study design (n = 48), not written or translated into English (n = 26), or having no response from authors where eligibility required clarification (n = 22). The RCT selection process is outlined in Fig 1.

### RCT characteristics

The characteristics of included RCTs are summarised in S1 Table. The 24 RCTs incorporated a total of n = 2366 participant from 10 countries. 18 RCTs recruited participants with knee OA [53–70], 3 RCTs recruited participants hip OA [71–73], and 3 RCTs [40,74,75] recruited participants with both knee and hip OA. In 21 RCTs all BCTs were delivered solely by physiotherapists. In 3 RCTs, the BCTs were delivered by both a physiotherapist (PA component) and either a nurse [67], health advisor [57], or researcher [68] delivering the telephone counselling. Training of BCT providers was not well reported, with 11 RCTs [59,60,62–70] not mentioning any training of the intervention provider and four RCTs [53,54,72,74] not reporting detail of the training duration. Training ranged from 3 hours [76] for instruction in an exercise intervention, to 4 days for pain coping skills training [56] and telephone behavioural counselling [57] respectively.

### PA adherence outcomes

Detail on PA and adherence outcomes used and the effect sizes at different time points within each domain is detailed in S2 Table. Analysis of outcome measure timings enabled short-term to be defined as up to 3 months (post-randomisation), medium-term approximately 6 months, and long-term 12 months or longer. The data closest to the temporal boundary of each time point was used to measure the effect sizes. 9 RCTs [54,60–62,64–66,69,70] did not measure outcomes beyond short term, with 7 RCTs evaluating outcomes up to medium-term [53,55,58,63,71,73,74] and 8 to long-term [40,56,57,67,68,72,75] respectively. Eight RCTs authors were contacted to provide additional data or clarify points, of which 5 [54,71–74] responded with additional data, 1 [64] clarified that PA was measured only at baseline, and 2 [40,68] could not be contacted to clarify information. Thirteen RCTs [40,54–57,61,67,69–74] measured PA adherence and contributed to BCT effectiveness ratio calculations. Therefore, despite using a BCT that promoted PA adherence as part of the behavioural physiotherapy intervention, 11 RCTs did not measure PA adherence between groups post baseline and therefore the effectiveness ratios could not be calculated. The BCTs identified in these RCT interventions were therefore used to demonstrate the breadth of BCT use and assist the narrative synthesis. PA adherence outcomes showed considerable heterogeneity between RCTs with 13 PA and 6 adherence measures identified. The quantity of PA adherence measures used in RCTs demonstrated further variability, with a range of 1 [54,61,70] to 6 [57,69]. Self-report PA measures included questionnaires, PA times and volumes, and direct PA measures included pedometers and accelerometers. Adherence measures included diaries/logbooks, self-rated PA adherence, and the number of PA sessions completed in the home/community.

### Risk of bias within and across RCTs

Six RCTs were assessed as low RoB [54,56,57,61,62,73] with five conducted by the same group of authors, suggesting some publication bias (Table 2). Seven and 12 RCTs were assessed as moderate [53,58,65,69,71,74,75] and high [40,55,59,60,63,64,66–68,70,72] RoB respectively. As meta-analysis was not indicated (see below), funnel plot use for meta-bias assessment was not appropriate [77]. Overall, there was high risk of performance bias with participant blinding assessed as either unclear or high RoB in 20 RCTs. Further internal validity issues included high risk of reporting and attrition bias with 10 (42%) and 8 (33%) RCTs at unclear or high RoB respectively.

**Table 2:** Summary of risk of bias assessment.

	Risk of Bias components						
Trial	1	2	3	4	5	6	7
Bennell 2005	L	L	L	L	L	U	L
Bennell 2010	L	L	H	L	L	L	L
Bennell 2014	L	L	H	H	L	L	L
Bennell 2014b	L	L	L	L	L	L	L
Bennell 2016	L	L	H	L	L	L	L
Bennell 2017	L	L	H	L	L	L	L
Crossley 2015	L	L	L	L	L	U	L
Deyle 2000	L	U	U	L	U	U	H
Dincer 2016	L	U	H	L	L	U	H
EMPART 2013	L	L	H	L	U	L	L
Hiyama 2012	L	L	H	L	L	U	H
Hunt 2010	L	L	L	L	L	L	L
Jones 2012	L	L	H	L	L	L	L
Kawasaki 2009	L	H	H	H	U	U	U
Kuru-Colak 2017	L	L	H	H	H	H	H
Lim 2008	L	L	H	L	L	U	L
MOA 2013	L	L	H	L	L	L	U
Odole 2013	L	H	H	H	U	U	H
Schlenk 2011	L	H	H	H	L	U	L
Segal 2015	L	L	H	H	U	L	H
Teirlinck 2016	L	L	H	H	U	L	L
Van Baar 1998	L	L	H	L	L	L	U
Veenhof 2006	L	H	H	L	L	L	U
Wallis 2017	L	L	H	L	U	L	U

### Fidelity

Overall the quality of reporting of fidelity components within domains was low (S3 Table). The mean component score per RCT was 38.9% (range 19% [66] to 68% [73]). No RCT was assessed as achieving ‘high fidelity’, which has been defined as having reported at least 80% of individual fidelity components clearly [43]. Furthermore, only 5 RCTs reported >50% of individual fidelity components clearly [56,57,61,73,75]. In particular, the theoretical aspects of intervention design and delivery, and practitioner training were poorly reported. However, 3 of the 5 domains were generally well met with all RCTs assessed to have reported enactment of skills, 23 RCTs receipt of treatment, and 20 RCTs treatment design satisfactorily. Overall the provider training and delivery of treatment domains were poorly reported with only 5 and 3 RCTs reporting them satisfactorily respectively.

#### BCTs identified in interventions

31 interventions (mean (SD): 11.1 (3.3)) were coded across the 24 RCTs (Table 3) with a total of 344 BCTs identified. Twenty-four interventions were classified as behavioural physiotherapy which interventions incorporated 269 BCTs (11.2 (2.9)), with a further 7 identified as active comparator groups which included 75 BCTs (10.71 (4.4)) respectively. The highest number of BCTs used in a single intervention was 20 [56] and the lowest 5 [40,70]. 31 distinct BCTs (including ‘a’ and ‘b’ subgroups) representing 32% of possible taxonomy definitions (n = 98) were identified and 12 out of 16 hierarchies were used. The most frequently identified BCTs were 8.6 generalisation of target behaviour (100% of interventions), 9.1 credible source (100%), 12.6 body changes (100%), 1.4 action planning (94%), 4.1 instruction on how to perform a behaviour (94%), and 8.1 behavioural practice/rehearsal (87%). The two independent researchers coded BCTs with ‘excellent’ inter-rater reliability (κ = 0.83).

**Table 3:** BCTs used in physiotherapy interventions of included RCTs.

	BCT LABEL	BEHAVIOURAL PHYSIOTHERAPY INTERVENTIONS																								ACTIVE COMPARATORS							N (%)
		1	2	3	4	5	6	7	8	9	10	11	12	13	14	15	16	17	18	19	20	21	22	23	24	25	26	27	28	29	30	31	
**1.1A**	**Goal Setting behaviour (behaviour)**					✓	✓																	✓			✓		✓				**5 (16)**
**1.1B**	**goal setting (behaviour)**	✓	✓	✓	✓	✓		✓	✓	✓	✓	✓			✓	✓	✓	✓	✓	✓	✓	✓	✓		✓	✓		✓					**22 (71)**
**1.2**	**Problem Solving**			✓		✓	✓						✓											✓			✓		✓				**7 (23)**
**1.3B**	**Goal setting (outcome)**																				✓		✓									✓	**3 (10)**
**1.4**	**Action planning**	✓	✓	✓	✓	✓	✓	✓	✓	✓	✓	✓	✓		✓	✓	✓	✓	✓	✓	✓	✓	✓	✓	✓	✓	✓	✓	✓	✓	✓		**29 (94)**
**1.5**	**Review behaviour goal (s)**						✓													✓													**2 (6)**
**1.8**	**Behavioural contract**																							✓									**1 (3)**
**2.1 A**	**Monitoring of behaviour by others without feedback**				✓	✓				✓									✓			✓			✓		✓	✓					**8 (26)**
**2.1 B**	**Monitoring of behaviour by others without feedback**												✓		✓		✓						✓								✓		**5 (16)**
**2.2**	**Feedback on behaviour**		✓	✓		✓							✓			✓				✓		✓		✓		✓	✓	✓			✓		**12 (39)**
**2.3A**	**Self-Monitoring of behaviour**	✓		✓		✓	✓	✓	✓		✓	✓						✓	✓	✓				✓	✓	✓	✓	✓	✓	✓			**18 (58)**
**2.3B**	**Self-Monitoring of behaviour**		✓		✓								✓	✓	✓		✓														✓		**7 (23)**
**2.6**	**Biofeedback**																				✓												**1 (3)**
**2.7**	**Feedback on outcomes of behaviour**																			✓	✓												**2 (6)**
**3.1A**	**Social Support (Unspecified)**					✓	✓	✓			✓					✓			✓	✓	✓			✓	✓		✓	✓	✓				**13 (42)**
**3.1B**	**Social Support (Unspecified)**				✓					✓													✓										**3 (10)**
**4.1**	**Instruction in how to perform the behaviour**	✓	✓	✓	✓	✓	✓	✓	✓	✓	✓		✓	✓	✓	✓	✓	✓	✓	✓	✓	✓	✓	✓	✓	✓	✓	✓	✓		✓	✓	**29 (94)**
**4.2**	**Information on Antecedents**					✓																					✓						**2 (6)**
**5.1**	**Information about health consequences**				✓		✓					✓																	✓				**4 (13)**
**5.3**	**Information about social and environmental consequences**						✓													✓									✓				**3 (10)**
**6.1**	**Demonstration of the behaviour**				✓	✓	✓	✓	✓									✓	✓	✓		✓		✓				✓	✓				**12 (39)**
**8.1**	**Behavioural Practice/ rehearsal**	✓	✓	✓	✓	✓	✓	✓	✓	✓	✓		✓	✓	✓	✓	✓	✓	✓	✓	✓	✓	✓	✓	✓		✓	✓	✓		✓		**27 (87)**
**8.6**	**Generalisation of the target behaviour**	✓	✓	✓	✓	✓	✓	✓	✓	✓	✓	✓	✓	✓	✓	✓	✓	✓	✓	✓	✓	✓	✓	✓	✓	✓	✓	✓	✓	✓	✓	✓	**31 (100)**
**8.7**	**Graded tasks**		✓		✓	✓	✓	✓	✓		✓		✓				✓	✓		✓				✓			✓	✓	✓		✓		**16 (52)**
**9.1**	**Credible Source**	✓	✓	✓	✓	✓	✓	✓	✓	✓	✓	✓	✓	✓	✓	✓	✓	✓	✓	✓	✓	✓	✓	✓	✓	✓	✓	✓	✓	✓	✓	✓	**31 (100)**
**10.3**	**Non-specific Reward**																							✓									**1 (3)**
**11.2**	**Reduce Negative Emotions**					✓							✓														✓						**3 (10)**
**12.4**	**Distraction**					✓							✓														✓						**3 (10)**
**12.5**	**Adding objects to the environment**	✓	✓	✓	✓	✓	✓							✓			✓									✓		✓	✓				**11 (35)**
**12.6**	**Body Changes**	✓	✓	✓	✓	✓	✓	✓	✓	✓	✓	✓	✓	✓	✓	✓	✓	✓	✓	✓	✓	✓	✓	✓	✓	✓	✓	✓	✓	✓	✓	✓	**31 (100)**
**15.4**	**Self-talk**					✓																					✓						**2 (6)**
**TOT**		**9**	**11**	**11**	**14**	**20**	**16**	**11**	**10**	**9**	**10**	**7**	**13**	**7**	**9**	**9**	**11**	**10**	**11**	**15**	**11**	**10**	**10**	**15**	**10**	**9**	**17**	**14**	**15**	**5**	**10**	**5**	**344**

**Key: TOT:** Total number of BCTs found in RCT interventions; N: Number of times BCT identified across interventions; %: Percentage of times BCT appears across interventions: Intervention number 1–24 represent behavioural physiotherapy interventions; numbers 25–31 represent active comparator groups.

Behavioural Physiotherapy Interventions

Intervention numbers

1) Bennell 2005 2) Bennell 2010 3) Bennell 2014 (PT + Booster) 4) Bennell 2014b 5) Bennell 2016 (PT + PCST) 6) Bennell 2017 (PT+ TP coaching 7) Crossley 2015 8) Deyle 20009) 9) Dincer 2016 10) EMPART 2013 (Both Ex + MT interventions 11) Hiyama 2012(PT +PA goal) 12) Hunt 2013 (PT + PCST) 13) Jones 2012 14) Kawasaki 2009 15) Kuru-Colak 2017 16) Lim 2008 17) MOA 2013 18) Odole 2013 19) Schlenk 2011 20) Segal 2015 21) Teirlinck 2016 22) Van Baar 1998 23) Veenhof 2006 (PT + BGA) 24) Wallis 2016

Active Comparators

25) Bennell 2014 (PT Control) 26) Bennell 2016 (PCST) 27) Bennell 2016 (PT control) 28) Bennell 2017 (PT control) 29) Hiyama(PT control) 30) Hunt 2013 (PT control) 31) Veenhof 2006 (PT control)

### BCT effectiveness

BCT effectiveness ratios calculated for short, medium and long-term PA adherence outcomes across domains are presented in Table 4. Overall, the BCTs with effectiveness ratios of 100% across all measured time frames were 1.8 behavioural contract, 2.7 Feedback on outcomes of behaviour, and 10.3 non-specific reward. Eight BCTs had a short-term effectiveness ratio of ≥50% in at least 2 outcome domains. Of these, 3 BCTs were from the goals and planning and 2 from the feedback and monitoring hierarchies respectively. Similarly, 8 BCTs had long-term effectiveness ratios of ≥50% with 4 BCTs coming from the goals and planning hierarchy. Medium-term effectiveness ratios were generally lower than short, or long-term measures. Adherence outcomes had higher proportions of effectiveness ratios of ≥50% than PA self-report or direct measures respectively. There was minimal long-term PA direct measure data available for analysis.

**Table 4:** BCT effectiveness ratios.

	OUTCOME DOMAIN/ TIMEFRAME								
	PA- Self Report			PA- direct measure			Adherence		
BCT	Short	Medium	Long	Short	Medium	Long	Short	Medium	Long
**1.1A**	1/2 (50)	0/1 (0)	1/2 (50)				1/2 (50)		1/2 (50)
**1.1B**	2/5 (40)	1/4 (25)	0/2 (0)	2/3 (67)	0/1 (0)		1/1 (100)	1/1 (100)	1/1 (100)
**1.2**	1/2 (50)	0/1 (0)	1/2 (50)				1/4 (25)	0/1 (0)	1/2 (50)
**1.3B**	0/1 (0)	0/1 (0)							
**1.4**	3/6 (50)	1/4 (25)	1/3 (33)	1/1 (100)	0/1 (0)	0/1 (0)	2/2 (100)	1/1 (100)	2/2 (100)
**1.5**		2/2 (100)	1/2 (50)		0/1 (0)			1/1 (100)	1/1 (100)
**1.8**	1/1 (100)		1/1 (100)				1/1 (100)		1/1 (100)
**2.1A**	0/2 (0)	0/2 (0)	0/1 (0)	1/2 (50)	0/1 (0)		1/1 (100)	1/1 (100)	1/1 (100)
**2.1B**	0/1 (0)	0/1 (0)							
**2.2**	1/2 (50)	1/2 (50)	0/2 (0)				1/1 (100)	1/1 (100)	1/1 (100)
**2.3A**	2/2 (100)	1/1 (100)	1/2 (50)	1/1 (100)			1/1 (100)		1/1 (100)
**2.3B**	1/2 (50)	0/1 (0)		0/1 (0)	0/1 (0)				
**2.7**		1/1 (100)	1/1 (100)						
**3.1A**	2/2 (100)	1/1 (100)	1/2 (50)				1/1 (100)		1/1 (100)
**3.1B**	0/2 (0)	0/2 (0)		0/1 (0)	0/1 (0)				
**4.1**	1/4 (25)	1/4 (25)	0/2 (0)	1/2 (50)	0/1 (0)		1/1 (100)	1/1 (100)	1/1 (100)
**4.2**	0/1 (0)	0/1 (0)	0/1 (0)				0/1 (0)		0/1 (0)
**5.1**	0/1 (0)	0/1 (0)		1/2 (50)	0/1 (0)				
**5.3**		1/1 (100)	0/1 (0)						
**6.1**	1/3 (33)	1/3 (33)	1/3 (33)	0/1 (0)	0/1 (0)		2/2 (100)	2/2 (100)	2/2 (100)
**8.1**	3/6 (50)	1/4 (25)	1/3 (33)	1/2 (50)	0/1 (0)		2/3 (67)	1/2 (50)	2/2 (100)
**8.6**	2/5 (40)	1/4 (25)	0/2 (0)	1/2 (50)	0/1 (0)		1/1 (100)	1/1 (100)	1/1 (100)
**8.7**	3/4 (75)	1/2 (50)	1/2 (50)	0/1 (0)	0/1 (0)		1/1 (100)		1/1 (100)
**9.1**	2/5 (40)	1/4 (25)	0/2 (0)	1/2 (50)	0/1 (0)		1/1 (100)		1/1 (100)
**10.3**	1/1 (100)		1/1 (100)				1/1 (100)		1/1 (100)
**11.2**	0/2 (0)	0/1 (0)	0/1 (0)				0/1 (0)		0/1 (0)
**12.4**	0/2 (0)	0/1 (0)	0/1 (0)				0/1 (0)		0/1 (0)
**12.5**	1/2 (50)	0/1 (0)		0/1 (0)	0/1 (0)				
**12.6**	2/5 (40)	1/4 (25)	0/2 (0)	1/2 (50)	0/1 (0)		1/1 (100)	1/1(100)	1/1 (100)
**15.4**	0/1 (0)	0/1 (0)	0/1 (0)				0/1 (0)		0/1 (0)
**TOTALS**	**12/15**	**7/21**	**9/15**	**10/5**	**0/15**	**0/1**	**16/5**	**10/ 1**	**18/4**

**Key:** BCT part of statistically effective intervention/BCT part of all interventions (effectiveness ratio %); Blank areas indicate where no PA adherence data was available for this domain/ timeframe

## Discussion

This is the first systematic review to measure the effectiveness of BCTs used within physiotherapy interventions to promote PA adherence in lower limb OA patients. The body of literature is defined by RCTs of medium to high RoB with poor intervention reporting. Further heterogeneity of BCTs and PA adherence outcome measures meant meta-analysis was not appropriate and limited the evidence-based recommendations that could be made.

The BCT taxonomy was found to have excellent inter-rater agreement (Kappa = 0.83) [46], which showed similar reliability to other systematic reviews where the coders undertook training, [16–20,22–25,27,34,51] (range 0.68–0.92) and the 31 BCTs identified across interventions (mean (SD): 11.2(2.9)) were consistent with the most closely related systematic reviews [27,28,52]. Most BCTs had effectiveness ratios of <50% across time-frames and outcome domains, meaning that they were components of interventions that were statistically no more effective than comparator groups at optimizing PA adherence. This overall lack of effect could be due to several reasons. Firstly, to be included, RCT interventions had to incorporate a BCT which would promote PA adherence when away from the clinic and possess at least one BCT which was distinct from its comparator group (when coded on the V1 taxonomy) to enable for its effectiveness ratio to be calculated. Despite this, of the 24 RCTs, only 13 reported PA adherence data between groups post baseline to enable effectiveness ratios to be calculated. This was reflected in the RCTs primary treatment aims objectives and outcome measures which generally focused on pain and function. The assessment of pain and function might not reflect changes in patient PA adherence as physical activity interventions generally aim to teach patients coping and self-management skills rather than trying to ameliorate their clinical symptoms [78].

Secondly, in order to determine individual BCT effectiveness on PA adherence, it was essential to code both the behavioural physiotherapy intervention and any active comparator groups. As several RCTs used physiotherapy comparison groups, the number of unique BCTs in the behavioural physiotherapy interventions that were used to calculate effectiveness ratios was reduced. Previous research has shown that greater numbers of unique BCTs are associated with increased effect sizes and therefore this may have contributed to the modest results [52].

Overall, five BCTs demonstrated effectiveness ratios of ≥50% in more than one outcome domain in both the short and long-time periods. These were 1.1A Patient led goal setting, 1.8 behavioural contract, 2.3A self-monitoring of behaviour, 3.1 A social support unspecified, and 10.3 non-specific reward. The most effective BCTs across time points were behavioural contract and non-specific reward (e.g. a reward was given to the patient if PA goals were met), both of which have been highlighted as effective in previous systematic reviews on PA [20,34]. However, these BCTs were only present in a single intervention in 1 high RoB RCT [40], therefore these results should be interpreted cautiously. The 3 further BCTs identified were the ‘a’ variations used specifically in this systematic review. Therefore, items which promote self-monitoring (e.g. activity diaries) and the provision of social support, which are explicitly intervention components may be more effective at promoting changes in PA adherence than if they are used for monitoring purposes only. This review showed that of the 26 interventions that incorporated the BCT goal setting (behaviour), only five reported it in a patient-led manner. Patients are more likely to adhere to health behaviours if their goals are related to activities they enjoy and fit with their values [79], therefore promoting intrinsically motivated autonomy and subsequently PA adherence [7].

Further BCTs that demonstrated high short -term effectiveness ratios were 1.4 action planning, 2.2 feedback on behaviour, 8.1 behavioural practice/rehearsal which have been shown effective on PA in other systematic reviews [16]. Overall, the BCTs that had the highest short-term effectiveness ratios were characterised as patient reminders to do their prescribed PA, either from the therapist directly (e.g. phone calls for monitoring purposes) or as physical items that were taken home (e.g. logbooks/diaries or handouts including detailed information about exercises). In the behaviour change literature, the initial period where patients learn new habits and health behaviours is termed the ‘adoption’ phase [7]. During adoption, new behaviours are enhanced by extrinsic factors, so the addition of BCTs that help form new habits likely optimised short-term PA adherence.

The period of longer-term behaviour change is commonly referred to as the ‘maintenance’ phase [79]. There are varying opinions in the literature about when a patient achieves maintenance, with authors using time scales or when the behaviour becomes the patients ‘dominant’ response [79] as key indicators. Interestingly the 6-month medium-term time period when BCT effectiveness ratios were reduced corresponds to the Transtheoretical model of changes’ [80] definition of maintenance, which is the most widely used in the behaviour change literature [79], and may explain the drop off in effectiveness at this point. Maintenance is theorised to require different skills and habits from adoption to prevent patients from relapsing to their previous behaviours [7]. However, most BCTs that had the highest effectiveness ratios at short-term follow up were the same techniques with the highest ratios at long-term time points. Further BCTs which demonstrated effectiveness ratios of ≥50% in at least two domains at long-term follow up were 1.2 problem solving, 1.5 review behaviour goals, and 8.7 graded tasks. Taken together, These BCTs are thought to be important at promoting self-regulation and habits (problem-solving, behavioural contract, self-monitoring of behaviour, graded tasks) or sustaining motivation (patient led goal setting, reviewing behaviour goals, non-specific reward, social support (unspecified)) which have been identified as critical factors that help maintain behavioural changes [79]. Therefore, the increase in BCT effectiveness ratios from medium to long-term follow up may be due to patients with lower limb OA overcoming their relapses using the identified BCTs. Although these techniques show promise, these results should be interpreted cautiously as the effectiveness ratios of these BCTs were generally modest and there was insufficient data to draw firm conclusions. However, they were the only techniques for which the ratios demonstrated effectiveness at both short and long-term follow ups and warrant further investigation in physiotherapy practice, especially their use and effect around the 6-month post baseline time period.

RCT intervention fidelity demonstrated poor reporting. Although the domains receipt of treatment and enactment of skills were judged present in all RCTs, the only other systematic review that has used the NIHBCC checklist [78] judged these to be much lower. The high percentage reported in this review is likely due to its focus on targeting PA adherence in the community and home as several components within these domains focused on the performance of the target behaviour and skills outside from the clinic. The delivery of treatment and provider training domains were identified as poorly reported in both reviews. 12 RCTs reported that some form of training or monitoring took place but only 8 reported any timing with the majority lacking detail to enable replication (S1 Table). Therefore, limited conclusions can be drawn on BCT effectiveness and replicating them in clinical practice is problematic [42]. Similarly to Toomey et al., (2015) [78], fidelity assessment demonstrated a lack of use of theoretical models of behaviour change used to underpin their interventions. Although several RCTs mentioned behavioural models [40,56,57,61], only one (Schlenk et al., 2011) [67] explicitly used theory to inform their intervention. Theoretically derived interventions are thought to be more effective than non-theoretical interventions at increasing patient adherence [31] as they address specific determinants of health behaviour to enable treatment effects to be attributed to intervention techniques and therefore further refinement over time [27]. However, Schlenk et al., (2011) [67] was assessed as high RoB and therefore, the results from this RCT must be viewed with caution. Interestingly, the BCTs that showed the most consistently high effectiveness i.e. patient-led goal setting [81], self-monitoring [82], and social support [83] are key constructs of several theories of health behaviour change. Therefore, as physiotherapists have demonstrated a lack of knowledge of theoretical models of behavioural change and confidence in putting them into practice [14], this represents a gap in the professional knowledge base and literature.

## Strengths and limitations

The strengths of this thoroughly conducted systematic review include its rigorous methodology conducted according to a published protocol. The key limitation was the limited number of RCTs that were characterised by poor intervention reporting and moderate to high RoB combined with heterogeneity of BCTs and PA adherence outcomes. This meant meta-analysis was not indicated, therefore limiting the evidence-based recommendations that could be made. Furthermore, despite several BCT effectiveness ratios showing statistically significant increases in PA and adherence, it was not possible to determine whether the results were clinically significant. As PA [84] and adherence [85] outcomes are measured heterogeneously, no standardised process exists to covert the data into clinical meaningful parameters. This remains a gap in the literature and requires further investigation.

## Conclusions

Overall, BCTs have limited effectiveness at promoting PA adherence as part of physiotherapy interventions with the medium term (6 months post baseline) the most difficult period to target. The BCTs patient-led goal setting, behavioural contract, self-monitoring of behaviour, social support (unspecified), and non-specific reward were the most effective at optimising PA adherence across time points. Future research should include low RoB and transparently reported RCTs incorporating physiotherapy interventions with pre-specified BCTs aimed at optimising PA adherence in patients with lower limb OA. The inclusion of interventions underpinned by behaviour change theory is a research priority.

## Supporting information

S1 Checklist PRISMA checklist. (DOC)

S1 Table Characteristics of Included RCTs. (DOCX)

S2 Table Physical activity and adherence outcomes in included RCTs. (DOCX)

S3 Table Fidelity domain assessment of included RCTs. (DOCX)
