# Supplementary material for: Effectiveness of behaviour change techniques in physiotherapy interventions to promote physical activity adherence in lower limb osteoarthritis patients: A systematic review
Source: PLoS One. 2019 Jul 10;14(7):e0219482. doi: 10.1371/journal.pone.0219482 (PMC6619772; doi:10.1371/journal.pone.0219482)
Supplement: S1 Table — (DOCX) [file pone.0219482.s002.docx]

**S1 Table: Characteristics of Included Trials**

|  | | Comparison Group | | Behavioural Physiotherapy intervention | | | Delivery and training | |
| --- | --- | --- | --- | --- | --- | --- | --- | --- |
| Trial /Country | **Type of OA** | **N, (M/F), Mean age** | **Content** | | **N, (M/F), Mean Age** | **Content and PA adherence BCT:** | **Delivery** | **Training in BCT delivery** |
| Bennell 2005, Australia | Knee | 67 (23/44), 69.8 | Sham Ultrasound, no detail on dose or timings | | 73, (23/50), 67.4 | 4 x 30-45 minute weekly sessions, followed by 4 x fortnightly sessions with the physiotherapist with HEP and taping provided. Log book given to measure HEP adherence. | Physiotherapist only | Trial mentions training and treatment manual given to physiotherapists by one researcher but no detail on timing or additional content |
| Bennell 2010, Australia | Knee | 44(24/20), 64.6 | No Intervention | | 45, (22/23), 64.5 | 5 weekly and 2 fortnightly (7 total) Physiotherapy sessions (30-minute initial and 15 minutes follow up) with HEP provided. Diary of home exercises (% of 60 days) to measure adherence | Physiotherapist only | Both protocol and trial mention training but no detail provided by whom, timing or content |
| Bennell 2014, Australia  (Original trial not included) | Knee | 38, (18/20), 63.7 | Participants asked to continue with HEP from original RCT – outcomes assessed via questionnaire at 24 weeks (mail out | | 40, (24/16), 60.5 | Control + 2 x individual booster sessions with physiotherapist with HEP adherence counselling and problem solving encouraged at weeks 8 and 16 | Physiotherapist only | 3-hour training during original RCT. |
| Bennell 2014b, Australia | Hip | 53, (17/36), 62.7 | Sham Ultrasound | | 49, (23/26), 64.5 | 10 sessions of 30-60 minutes over 12 weeks with HEP provided. Log book given to measure HEP adherence. Phone calls to monitor adherence (treatment manual) | Physiotherapist only | 2-day workshop with treatment manual and DVD provided |
| Bennell 2016, Australia | Knee | PSCT:  74, (45/29), 63.0;  Exercise:  75, (44/31), 62.7; | PSCT:  10 x 45-minute sessions with physiotherapist over 12 weeks; Sessions were PCST and training and homebased practice encouraged, Physiotherapists telephoned patients to discuss progress and adherence in weeks 22,38 and 46.  Exercise:  10 x 25-minute sessions with physiotherapists over 12 weeks. HEP given to patients. Physiotherapists telephoned patients to discuss progress and adherence in weeks 22,38 and 46 | | 73, (44/29), 64.6; | 10 x 70-minute sessions with physiotherapists over 12 weeks teaching both HEP and PCST to patients. Physiotherapists telephoned to discuss progress and adherence to the home program in weeks 22, 38 and 46. | Physiotherapist only | PCST: Psychologist led 4-day group workshop, followed by group tutorials and individual practice, feedback provided from Audio recordings  Exercise: 4-hour workshop with treatment manual and DVD of workshop provided |
| Bennell 2017, Australia | Knee | 84, (27/57),61.1 | 5 x 30-minute sessions over 6 months with physiotherapist focusing on HEP and education. Log book and Pedometer given to patient to monitor adherence. | | 84, (35/29), 63.4 | Physiotherapy control + 6-12 telephone-delivered coaching sessions (30-45-minute initial; 15-30 minute follow up) with nurse over 6 months to encourage adherence to PA and adherence behaviours | Physiotherapists: Exercise component  Health coach (nurse, occupational therapist, psychologist): Telephone component | Physiotherapists: One-day training and provision of treatment manual  Health coaches: One-day training of health coaches with no experience had additional 3 day ‘Health change Australia’ training with lead researcher. Practice sessions between days 2-3 of course with one face to face session with lead researcher and 3 practice patients provided. Initial 2 patients reviewed and feedback given. Further feedback as needed. |
| Crossley 2015, Australia | Knee | 48, (19/29), 53 | 8 x 60-minute education sessions delivered with physiotherapist | | 44, (20/24), 56 | 8 x 60-minute (1/week for 4 weeks, then 1/week fortnightly for 8 weeks) Physiotherapy sessions with HEP, manual therapy and taping. Log book to monitor adherence to HEP. | Physiotherapist only | 6 hours of training provided to physiotherapists by lead researcher and treatment manual given. Monitoring mentioned but no detail provided |
| Deyle 2000, USA | Knee | 36, (18/18), 62.4 | Sub therapeutic ultrasound. 8 x 30minute sessions (2/ week for 4 weeks) with physiotherapists | | 33, (12/21), 59.6 | 8 x 30-45-minute sessions (2/ week for 4 weeks) with physiotherapist with HEP. Logbook given to monitor HEP adherence. | Physiotherapist only | No detail on training provided |
| Dincer 2016, Turkey | Knee | 16, (3/11),52.9, | 15 sessions x 30 minutes (5/ week for 3 weeks) with physiotherapist providing TENS and hot-pack | | 19, (3/13), 49.5 | Placebo + 15 sessions x 30 minutes (5/ week for 3 weeks) with physiotherapist, with HEP followed by 9 x weeks of home exercises. Adherence checked by phone fortnightly | Physiotherapist only | No detail on training provided |
| EMPART (French) 2013, Ireland | Hip | 43 (20/23), 60.8 | Waitlist control | | Group 1:  Exercise Physiotherapy  45 (11/34), 61.8  Group 2:  Exercise and manual Physiotherapy:  43(16/27), 64.8 | Group 1: Exercise Physiotherapy  6 – 8 x 30 min sessions over 8 weeks with physiotherapist. HEP taught. Activity and home exercise diaries to monitor adherence.  Group 2: Exercise and manual Physiotherapy:  6-8 x 45-minute sessions over 8 weeks with physiotherapist including 30 min of exercise therapy and up to 15 min of manual therapy; Activity and home exercise diaries to monitor HEP adherence. | Physiotherapist only | Training in exercises and manual therapy provided in 2 sessions by lead researcher and treatment manual given to participating therapists. No detail on length of sessions. |
| Hiyama 2012, Japan | Knee | 20, (0/20), 73.8 | 4 weekly sessions including exercises and cryotherapy | | 20, (0/20), 71.9 | Physiotherapy control plus PA walking goal set. | Physiotherapist only | No detail on training provided |
| Hunt 2013, Australia | Knee | 10, (NS), 59.8 | 10 x 60-minute sessions with physiotherapist with HEP plus NDC. | | 10, (NS), 66; | 10 x 60 minute weekly sessions with physiotherapist focusing on HEP plus PCST training to change patients PA behaviours. | Physiotherapist only | PCST: Psychologist led two-day training workshop with follow up tutorials and practice with feedback. Physiotherapists had to meet written criteria prior to accreditation.  NDC plus exercise: one-day training session with practice integrated into session. |
| Jones 2012, Brazil | Knee | 32, (NS: 11/89 total) 62.6 | Control, no cane intervention – instructed to use no gait devices | | 32, (NS: 11/89 total) 61.8 | 1 x 5-minute physiotherapy session for provision on Cane and education on its use. Adherence to cane use in intervention group documented via chart | Physiotherapist only | No detail on provider training given |
| Kawasaki 2009, Japan | Knee | 42, (0/42), 69.5 | 9 Hyaluronate injections (1/week for 5 weeks; 1 / month for 4 months). | | 45, (0/45), 71.2 | 2 sessions (initial plus 1 month) with Physiotherapist instructed on HEP. Diary measured adherence to HEP. | Physiotherapist only | No detail on provider training given |
| Kuru-Colak 2017, Turkey | Knee | 23, (9/24), 60  . | 18 sessions of 40-45 mins (3/ week for 6 weeks) focused on HEP | | 23, (15/8), 59 | Home based exercise programme; 1 x physiotherapy session where HEP taught. Weekly Follow up telephone call to monitor adherence for 6 weeks. | Physiotherapist only | No detail on provider training given |
| Lim 2008, Australia | Knee | 54 (25/29), 63.6 | No intervention | | 53, (23/30), 65.6 | 7 sessions over 10 weeks with a physiotherapist focused on HEP. Diary measured HEP adherence. | Physiotherapist only | No detail on provider training given |
| MOA (Abbott) 2013, New Zealand | Hip and Knee | 51, (26/25), 66.1 | No Intervention | | Group 1:  Exercise physiotherapy  51 (19/32), 66.9  Group 2:  Exercise physiotherapy plus Manual Therapy  50, (21/29), 66.0 | Group 1:  Exercise physiotherapy  11 x 50-minute sessions (1 /week for 9 weeks + 2 booster sessions at week 16) focused on HEP. Diary measured HEP adherence.  Group 2:  Exercise physiotherapy plus Manual Therapy  11 x 50-minute sessions (1 /week for 9 weeks + 2 booster sessions at week 16) focused on HEP with addition as manual therapy. Diary measured HEP adherence. | Physiotherapist only | 3 days of training by lead researcher including treatment protocol, monthly team meetings and feedback |
| Odole 2013, Nigeria  (includes Odole^1^) | Knee | 25, (14/11), 55 | 18 sessions (3 x week for 6 weeks) with physiotherapist focused on exercise in clinic only. | | 25, (14/11), 56 | 1 x Physiotherapy session with Physiotherapy focused on HEP. 18 sessions of Tele-physio (3 / week for 6 weeks) exercise log book given to monitor HEP adherence. | Physiotherapist only | No detail on provider training given |
| Schlenk 2011, USA | Knee | 13, (NS Overall: 1/25) NS Overall: 63.2 | 1 session with physio at end of 6 months intervention phase. | | 13, (NS Overall: 1/25) NS Overall: 63.2 | 6x 60-minute physiotherapy sessions physio focussing on HEP and 9 fortnightly phone calls (15-30 Mins) with registered nurse focusing on PA adherence and behaviour change. PA diary given to monitor PA adherence and reviewed during telephone consults. | Exercise component: Physiotherapist  Telephone Component: Nurse | No detail on provider training given |
| Segal 2015, USA | Knee | 19 (9/10),69.6 | Arthritis Foundation symptom diary | | 29, (7/22), 69.1 | 24 x 45 min (2/ week for 12 weeks) sessions with physio focused on walking education. 4 x telephone motivational interviewing counselling sessions with physiotherapist (months 4,5,8 and 10) to monitor and encourage PA adherence. | Exercise component: Physiotherapist  Telephone Component: Not clear (‘researcher’) | No detail on provider training given |
| Teirlinck 2016, The Netherlands | Hip | 102, (46/56), 67 | Usual care given by patients GP and brochure about hip OA | | 101, (38/63),64 | UC + 12 x 30-minute sessions over 3 months with Physiotherapist focusing on HEP. 3 x Booster sessions in month 5, 7, 9. Physiotherapist recorded PA adherence at each session. | Physiotherapist only | Training at consensus meeting of Dutch physiotherapy guide lines and treatment manual mentioned but no detail on length of training or by whom. |
| Van Baar 1998, The Netherlands  (Includes Van Baar, 2001) | Hip and Knee | 102, (21/81),67.7 | GP only | | 98, (22/76),68.3 | UC + Physiotherapy sessions: Up to 3/ week for 12 weeks (30-minute duration) focused on HEP. Telephoned to check adherence to HEP in weeks 6 and 12. | Physiotherapist only | 2 training sessions in protocol given. Exercises reviewed and practiced. No detail on length of sessions or who provided training (assumed lead researcher based on protocol development). |
| Veenhof 2006, The  Netherlands (Includes Pisters, 2010^1, 2,3^) | Hip and Knee | 103, (22/81),64.5 | 18 x 30-minute sessions over 12 weeks with a physiotherapist focused on HEP based on the Dutch physiotherapy guidelines. | | 97, (24/73),65.1 | 18 x 30-minute sessions over 12 weeks with 5-7 x 30-minute booster sessions between weeks 18-55 with physiotherapist. Sessions based integrated operant conditioning and exercise therapy focusing on PA adherence. | Physiotherapist only | Behavioural Physiotherapy: 2-day training with supervision via telephone throughout trial  Control Physio: 4-hour workshop on Dutch guidelines. No details on who provided training. |
| Wallis 2017, Australia | Knee | 23, (12/11), 67 | No intervention | | 23, (14/9), 68  . | 1x 30-minute session with a physiotherapist to plan walking location/ time/ duration. Weekly session with Physiotherapist to monitor walking. SMS and telephone monitoring with physiotherapist. Pedometer and log book given to monitor PA adherence. | Physiotherapist only | No detail on provider training given |

**Key:**

BCT: Behaviour Change Technique ; OA: Osteoarthritis ; ACR: American College of Rheumatology; HEP: Home exercise programme; RCT: Randomized controlled trial; PCST: Pain Coping Skills Training; BL: Baseline ; NDC: Non-directive counselling.
