# Supplementary material for: Effectiveness of behaviour change techniques in physiotherapy interventions to promote physical activity adherence in lower limb osteoarthritis patients: A systematic review
Source: PLoS One. 2019 Jul 10;14(7):e0219482. doi: 10.1371/journal.pone.0219482 (PMC6619772; doi:10.1371/journal.pone.0219482)
Supplement: S2 Table — (DOCX) [file pone.0219482.s003.docx]

**S2 Table: Physical Activity and Adherence outcomes in Included Trials**

|  | Outcome Measure (PA/ adherence measure domain) | Outcome Timings / Effect sizes (Continuous outcome; SMD (95% CI); Dichotomous; PR (95% CI)) | | |
| --- | --- | --- | --- | --- |
| Trial |  | Short | Medium | Long |
| Bennell 2005 | Adherence:   - Diary to measure HEP done in intervention group. Not measured between groups. | 12 Weeks | 24 Weeks | NM |
| Bennell 2010 | Physical Activity (self-report):   - PASE   Adherence:   - Diary to measure percentage of HEP (n/60) done in intervention group. Not measured between groups. | 13 weeks  **-0.49 [-0.94, -0.03]+** | NM | NM |
| Bennell 2014 | Adherence:   - **Patient report of home exercises in previous week (%) (week 16)** - **11 -point NRS over previous 8 weeks (Week 24)** | 16 weeks  -0.14 [-0.58, 0.31]+ | 24 weeks  -0.60 [-2.09, 0.89]+ | NM |
| Bennell 2014b | Physical activity (self-report):   - **PASE**   Physical Activity Recorded (direct measure):   - **Steps per day (Pedometer)**   Adherence:   - Adherence to home exercises (%). Not measured between groups | 13 weeks  -0.07 [-0.47, 0.33]+  0.16 [-0.25, 0.56]+ | 36 weeks  -0.15 [-0.58, 0.29]+  -0.19 [-0.62, 0.24]+ | NM |
| Bennell 2016 | Physical Activity (self-report):   - **PASE**   Physical Activity (direct measure):   - Pedometer (NR)   Adherence:   - **Adherence to home exercises (%) reported in log books** - Self-rating on NRS at during treatment and average throughout 52 weeks follow up - Therapist rated adherence during treatment phase | 12 weeks  -0.07 [-0.41, 0.26]+  0.30 [-0.04, 0.64]+ | 22 weeks  -0.36 [-0.72, -0.00]+ | 52 weeks  -0.10 [-0.45, 0.25]+  -0.11 [-0.47, 0.25]+ |
| Bennell 2017 | Physical Activity (self-report):   - **PASE** - AAS   Physical Activity (direct measure):   - **Total no of steps per day (accelerometer)** - No of step hours / day   Adherence:   - **HEP sessions completed in past 2 weeks in log book (%):** - Self-rated adherence to home exercise (NRS) - Therapist rated home exercises | NM | 6 months  **-0.41 [-0.74, -0.08]+**  -0.20 [-0.53, 0.13]+  **-1.97 [-2.38, -1.57]+** | 12 months  -0.08 [-0.41, 0.26]+  **-0.42 [-0.76, -0.08]+** |
| Crossley 2015 | Adherence:   - Diary to measure HEP done in intervention group only. Not measured between groups | 3 months | 9 months | NM |
| Deyle 2000 | Adherence:   - Diary to measure HEP done in intervention group only. Not measured between groups | 8 weeks | NM | 1 year |
| Dincer 2016 | Adherence:   - Diary to measure HEP done in intervention group only. Not measured between groups | 12 weeks | NM | NM |
| EMPART 2013 | Physical Activity (self-report):   - **IPAQ Short Form**   Adherence:   - Diary to measure HEP done in intervention group only. Not measured between groups | 9 weeks  **-0.44 [-0.81, -0.07]+** | NM | NM |
| Hiyama 2012 | Physical Activity (direct measures):   - **Step count (pedometer)** | 4 weeks  **-1.96 [-2.73, -1.19]+** | NM | NM |
| Hunt 2013 | Adherence:   - **HEP recorded in log book (%)** | 11 weeks  -0.02 [-0.92, 0.88]+ | NM | NM |
| Jones 2012, Brazil | Adherence:   - Cane use as walking aid recorded in log book. Not measured between groups. | 60 days | NM | NM |
| Kawasaki 2009, Japan | Adherence:   - HEP recorded in log book and monitored in treatment sessions. Not measured between groups | NM | 24 weeks | NM |
| Kuru-Colak 2017, Turkey | Physical Activity:   - IPAQ (in methods but not reported)   Adherence:   - Intervention group received telephone calls to monitor compliance to HEP. Not measured between groups. | 6 weeks | NM | NM |
| Lim 2008 | Adherence:  HEP recorded in log book (%). Not measured between groups. | 13 weeks | NM | NM |
| MOA 2013 | Adherence:  HEP recorded in log book in intervention group. Not measured between groups. | 9 weeks | 6 months | 1 year |
| Odole 2013 | Adherence:  HEP recorded in log book in intervention group only. Not measured between groups. | 6 weeks | NM | NM |
| Schlenk 2011 | Physical Activity (Self-Report):   - **Total volume exercise per week by diary** - Participation in fitness walking and other aerobic physical activities by diary | NM | 6 months  **-1.10 [-2.03, -0.16]+** | 11 months  -0.83 [-1.73, 0.07]+ |
| Segal 2015 | Physical Activity:   - PASE only done at baseline. Not measured between groups during or post intervention | 3 months | 6 months | 12 months |
| Teirlinck 2016 | Physical Activity (self-report):   - **Minutes per day of PA** - Days per week of PA   Adherence:   - **Self-report of whether doing prescribed PA over past 3 months (dichotomous)** | 3 months  0.10 [-0.31, 0.51]+   - 1. **[1.79, 3.26]%** | 6 months  -0.28 [-0.70, 0.15]+  **2.73 [2.01, 3.71]%** | 12 months  0.13 [-0.31, 0.57]+  **2.80 [1.87, 4.19]%** |
| Van Baar 1998 | Physical Activity (Self-report):   - **Zutphen PAQ** - Number who improved in each group (dichotomous)   Adherence:   - Patient and physiotherapist reported on 5-point scale then dichotomised. Not measured between groups. | 1. Weeks   0.24 [-0.04, 0.53]+ | 36 weeks  0.20 [-0.09, 0.49]+ | NM |
| Veenhof 2006,  (Includes Pisters, 2010^1, 2,3^) | Physical Activity (self-report):   - **Number of days per week moderate to vigorous physical activity (Pisters^1^)**   Adherence:   - **Self-rated home exercises 5-point scale. 4-5 classified as yes; 1-3 as no. Results then dichotomised** - Self-rated home exercises 5-point scale. 4-5 classified as yes; 1-3 as no. Results then dichotomised | 1. Weeks   **-1.40 [-2.11, -0.69**]+  **1.66 [1.24, 2.21]%** | 39 weeks | 65 weeks  **-1.40 [-2.21, -0.59]+**  **1.75 [1.20, 2.54]%** |
| Wallis 2017 | Physical Activity (direct measures):   - **Steps per Day** - Time walking > 80 steps/min per week (Moderate Intensity) - Minutes walked per day | 1. Weeks   **-0.83 [-1.48, -0.19]+** | NM | NM |

**Key:**

SMD: Standardised Mean Difference for continuous data; PR: Probability ratio for dichotomous data; CI: Confidence interval; NM: Not measured; Measure in each domain that was written in bold was used to determine effect sizes; NR: No results available; Results in bold show statistically significant effect in favour of behavioural physiotherapy intervention (*P*< 0.05); No results were able to be calculated if Physical activity or adherence was not measured between groups; HEP: Home exercise programme; PASE: Physical Activity Scale for the Elderly; AAS: Active Australia Survey; IPAQ: International Physical Activity Questionnaire; ZPAQ: Zutphen Physical Activity Questionnaire; SQUASH: Short Questionnaire to Assess Health Enhancing Physical Activity; NRS: Numerical Rating Scale; + indicates continuous data; % indicates dichotomous data
