# Supplementary material for: Effectiveness of behaviour change techniques in physiotherapy interventions to promote physical activity adherence in lower limb osteoarthritis patients: A systematic review
Source: PLoS One. 2019 Jul 10;14(7):e0219482. doi: 10.1371/journal.pone.0219482 (PMC6619772; doi:10.1371/journal.pone.0219482)
Supplement: S3 Table — (DOCX) [file pone.0219482.s004.docx]

**S3 Table: Fidelity Domain Assessment of included RCTs**

|  | Domains | | | | | Components |
| --- | --- | --- | --- | --- | --- | --- |
| Trial | Treatment Design | Training Provided | Delivery of Treatment | Receipt of Treatment | Enactment of skills | % of individual components present |
| Bennell 2005 | A | A | A | P | P | **33** |
| Bennell 2010 | P | A | A | P | P | **28** |
| Bennell 2014 | P | A | A | P | P | **44** |
| Bennell 2014b | P | P | P | P | P | **68** |
| Bennell 2016 | P | P | P | P | P | **66** |
| Bennell 2017 | P | P | A | P | P | **62** |
| Crossley 2015 | P | A | A | P | P | **37** |
| Deyle 2000 | P | A | A | P | P | **33** |
| Dincer 2016 | A | A | A | P | P | **21** |
| EMPART 2013 | P | A | A | P | P | **41** |
| Hiyama 2012 | P | A | A | A | P | **21** |
| Hunt 2013 | P | P | A | P | P | **55** |
| Jones 2012 | P | A | A | P | P | **28** |
| Kawasaki 2009 | P | A | A | P | P | **36** |
| Kuru-Colak 2017 | P | A | A | P | P | **30** |
| Lim 2008 | P | A | A | P | P | **28** |
| MOA 2013 | P | P | P | P | P | **63** |
| Odole 2013 | A | A | A | P | P | **19** |
| Schlenk 2010 | P | A | A | P | P | **41** |
| Segal 2015 | p | A | A | P | P | **28** |
| Teirlinck 2016 | P | A | A | P | P | **44** |
| Van Baar 1998 | P | A | A | P | P | **32** |
| Veenhof 2006 | A | A | A | P | P | **43** |
| Wallis 2017 | P | A | A | P | P | **32** |
| Total (N/24) | **20** | **5** | **3** | **23** | **24** | **MCP 38.9 SD:14.1** |

**Key: MCP- Mean components present; P- Present; A- Absent**
